# Neuronal MHC-I expression and its implications in synaptic function, axonal regeneration and Parkinson’s and other brain diseases

**DOI:** 10.3389/fnana.2014.00114

**Published:** 2014-10-13

**Authors:** Carolina Cebrián, John D. Loike, David Sulzer

**Affiliations:** ^1^Department of Neurology, Columbia University Medical CenterNew York, NY, USA; ^2^Department of Physiology and Cellular Biophysics, Columbia University Medical CenterNew York, NY, USA; ^3^Departments of Psychiatry and Pharmacology, Columbia University Medical CenterNew York, NY, USA

**Keywords:** major histocompatibility complex class I, neurons, neuroinflammation, neurodegeneration, plasticity

## Abstract

Neuronal expression of major histocompatibility complex I (MHC-I) has been implicated in developmental synaptic plasticity and axonal regeneration in the central nervous system (CNS), but recent findings demonstrate that constitutive neuronal MHC-I can also be involved in neurodegenerative diseases by playing a neuroinflammtory role. Recent reports demonstrate its expression *in vitro* and in human postmortem samples and support a role in neurodegeneration involving proinflammatory cytokines, activated microglia and increased cytosolic oxidative stress. Major histocompatibility complex I may be important for both normal development and pathogenesis of some CNS diseases including Parkinson’s.

## Introduction

The major histocompatibility complex (MHC) gene family encodes molecules on the surface of cells that enable the immune system to recognize presented self- and foreign-derived peptides (Chemali et al., 2011). The MHC genes (in human, HLA-A, -B, -C and HLA-DP, -DM, -DOA, -DOB, -DQ, -DR; in mouse, H-2-K, -D, -L, and 2-I-A and I-E allomorphs) are generally divided into three categories: class I, II or III. Class I MHC (MHC-I) molecules are expressed by nearly every mammalian cell class, while MHC class II (MHC-II) molecules are restricted to cells of the immune system, such as macrophages and lymphocytes. In human, genes encoding for MHC-I and MHC-II have a large number of alleles, leading to a great diversity of sets of MHC molecules in our species. MHC class III genes code for other immune system proteins, including components of the complement system and proinflammatory cytokines, as well as proteins not involved in immune function (Janeway et al., 2001).

**Figure 1:**
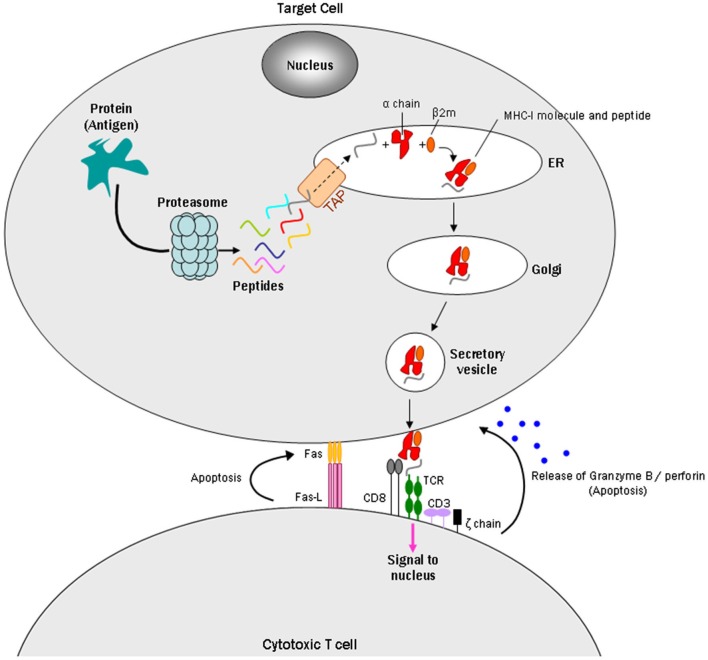
**Model of a CD8+ cytotoxic T cell targeting an MHC-I expressing cell**. Intracellular antigens are digested by the proteasome into small peptides. A specialized carrier, the *transporter associated with antigen processing* (TAP) complex, translocates the peptide into the endoplasmic reticulum (ER), allowing the antigen to bind MHC class I, which consists of an alpha and beta 2 microglobulin (β2m) chain. Endoplasmic reticulum-derived vesicles that contain the complex fuse with the Golgi. The complex is then packaged in secretory vesicles that fuse with the plasma membrane to insert the complex on the cell surface. Major histocompatibility complex I molecules present antigens to CD8^+^ cytotoxic T cells. The T cell CD8^+^ molecule recognizes MHC-I, while TCRs recognize specific antigenic peptides. The TCR complex contains CD3 and ζ(zeta) chains. Once the CD8^+^ T cell recognizes the target cell, apoptosis can occur in two ways: one uses the Fas ligand protein, which is expressed on the surface of the CD8^+^ T cells and binds to the Fas receptor on the target cell, which triggers apoptosis through the classical caspase cascade. The other uses secretion of granzyme B and perforin from T cell granules into the intercellular space between the cells.

MHC-I consists of two non-covalently linked polypeptide chains, known as alpha (α) and beta 2 microglobulin (β2m) chains (Cresswell et al., 2005). The complex can bind a large set of antigenic peptide fragments derived from degradation of intracellular proteins by the proteasome, which requires the “transporter associated with antigen processing” (TAP; Van Kaer et al., 1992). The MHC-I/peptide complex is then transferred to a vesicle that fuses with the plasma membrane to present the peptide fragment extracellularly. These antigens can then be identified by cytotoxic T lymphocytes (CTLs) or natural killer cells as “self” or “nonself” peptides, which leads to various responses depending on their receptors (Fleischer et al., 1986; Pawelec et al., 1986). If CTLs recognize the peptides as non-self antigens, they kill the presenting cells through the Fas or perforin pathways and/or indirectly by the release of cytokines (Andersen et al., 2006; Figure 1).

It has generally been presumed that the central nervous system (CNS) is immune-privileged and that MHC-I is not expressed by neurons (Lampson, 1995). However, accumulating data have demonstrated MHC-I expression by subsets of neurons in both adult and developing mammalian brain (for review, see Cullheim and Thams, 2010). Many of these reports describe a role for neuronal MHC-I in synaptic plasticity, brain development and axonal regeneration. Recent studies suggest that neuronal expression of this molecule is involved in neuroinflammatory processes and participates in immune-mediated neurodegeneration. In particular, there have been many reports linking neuroinflammation and Parkinson’s disease (PD; Tansey and Goldberg, 2010), and new data from our group suggests that expression of MHC-I by substantia nigra (SN) and locus coeruleus (LC) neurons may be involved in these inflammatory processes (Cebrián et al., 2014).

This review summarizes the pattern of expression and the different implications of neuronal MHC-I in the brain, and focuses in particular in the potential role of constitutive MHC-I expression by specific subsets of neurons in neurodegenerative diseases such as PD.

## Pattern of MHC-I expression in CNS neurons

Although the presence of MHC-I in the mature rodent CNS was for many years thought to be confined to glial cells (Wong et al., 1984), ensuing reports demonstrate MHC-I expression by some neuronal populations, both *in vitro*, usually triggered by exposure to interferon gamma (IFN-γ), and *in vivo*. The initial such study showed that MHC-I genes expression were induced by IFN-γ in cultured rat hippocampal neurons (Neumann et al., 1995).

Subsequently, mRNA for MHC-I was identified in nuclei of neonatal and adult rodent brain including the SN, brainstem motor neurons (Lindå et al., 1999), lateral geniculate nucleus (LGN), cortex, hippocampus (Huh et al., 2000) and cerebellum (Letellier et al., 2008). In aged rat motoneurons, mRNA for MHC-I and β2m increased with age (Edström et al., 2004).

Multiple groups reported expression of MHC-I subunits by immunolabel in CNS regions including cingulate cortex and hippocampus (Needleman et al., 2010; Liu et al., 2013), with expression gradually decreasing as neonatal mice reached adulthood (Liu et al., 2013). A recent study shows that MHC-I proteins are widely expressed in the developing mouse CNS at mid-gestation (E9.5–10.5), including the neuroepithelium and olfactory placode (Chacon and Boulanger, 2013).

In human brain, MHC-I expression was initially reported in microglia and endothelial cells of the hippocampus in control individuals and Alzheimer’s disease patients (Tooyama et al., 1990), but not in neurons. In contrast, MHC-II immunolabeling microglia, but not neurons, was shown in the SN of patients with Alzheimer’s disease and PD (McGeer et al., 1988), and in the hippocampus of patients with dementia with Lewy bodies (Imamura et al., 2005).

The neuronal expression of MHC-I in human brain has to date only been reported in a few studies. The first was a study of a childhood viral infection, Rasmussen’s encephalitis, in which immunolabel for the MHC-I component, β2m, was present in cortical and hippocampal neurons (Bien et al., 2002); more recently, MHC-I immunolabel was observed in dysmorphic/dysphasic cortical neurons of focal cortical dysplasia, tuberous sclerosis complex and ganglioglioma cases (Prabowo et al., 2013).

Most of the reports on MHC-I expression in human CNS neurons have been restricted to early development. In the embryo, β2m immunolabel was observed at 29–31 gestational weeks in the LGN of the dorsal thalamus, but was nearly absent by postnatal day 55, and was completely absent in the adult (Zhang et al., 2013b). In the human visual cortex, MHC-I was not observed at any gestational or postnatal stage (Zhang et al., 2013b), while the expression of MHC-I was very low in the hippocampus at 20 gestational weeks and slowly increased during weeks 27–33. A rapid increase in MHC-I molecule expression was found in the subiculum that reached high levels at 31–33 gestational weeks, but no expression of MHC-I was found in the adult hippocampus (Zhang et al., 2013a).

Several neurodegenerative disorders including PD are well established to display neuroinflammatory components and neuronal death (Tansey and Goldberg, 2010). As MHC-I is involved in antigen presentation and cell death (Chemali et al., 2011), we investigated its role in the degeneration of catecholamine neurons that are targeted in PD. Using immunolabel, mass spectroscopy, and mRNA analysis from laser captured neurons of adult control individuals and PD patients (Cebrián et al., 2014), we found that MHC-I is expressed by SN dopaminergic (DA) and LC norepinephrinergic (NE) neurons. Further analysis of isolated neuromelanin (NM) from these neurons by mass spectroscopy identified specific HLA alleles of MHC-I recovered from SN neurons, providing means to genotype the HLA type from neurons in postmortem human brain. Most of the immunolabel appeared to be present in NM, which are contained in modified autophagic lysosomes, but membrane preservation in postmortem human tissue is too poor to clearly ascertain whether the MHC-I is actually present on the plasma membrane at the time of death.

Our data from human tissue were supported by *in vitro* experiments that show DA human neurons derived from human embryonic stem cells normally do not express MHC-I but will do so following exposure to IFN-γ. Cultured primary catecholamine murine neurons also normally do not express MHC-I, but do so upon exposure to IFN-γ, activated microglia or exposure to high levels of L-dihydroxyphenylalanine (L-DOPA), and are far more susceptible to MHC-I induction than other neuronal populations tested, including cortical, striatal and thalamic neurons (Cebrián et al., 2014). These findings suggest that neuronal MHC-I expression and antigen display in catecholamine neurons may be triggered by microglial activation or high cytosolic DA, which in the presence of the appropriate antigen and CTLs could play a role in neuronal death during diseases in which CNS inflammation is robust. Thus, these results suggest reason to further explore roles for activated microglia, antigen presentation, neuronal MHC-I expression and recruitment of CTLs in neurodegenerative diseases, including PD, that feature the presence of T cells, activated microglia, intracellular oxidative stress and aggregates of alpha-synuclein (α-syn) in the SN and LC. These data may set a stage for understanding selective CTLs/MHC-I mediated neurodegeneration and set the basis for redefining the immunological component of PD, as well as provide evidence for a novel mechanism of neuronal death due to T-cell activity on which new therapies and treatments could be based.

## Roles for MHC-I in brain development and synaptic plasticity

It is well established that developing neurons express MHC-I (Shatz, 2009). A phenomenon implicated in development and maintenance of neuronal circuitry in the visual system (for review see Higenell and Ruthazer, 2010), the hippocampus, the cerebellum and the cortex (Ribic, 2012). Major histocompatibility complex I expression by developing neurons may be involved in retrograde signaling that regulates synaptic structure (Goddard et al., 2007). Neuronal MHC-I signaling may in some cases require regulators of cellular differentiation (Potthoff and Olson, 2007) known as myocyte enhancer factor 2 transcription factors (Elmer et al., 2013) to eliminate synapses during brain development.

In the visual system, mice deficient for MHC-I or the T cell receptor (TCR) subunit, CD3zeta (also known as CD247), exhibit reduced retinal synaptic activity, incomplete developmental refinement of connections between retina and its central targets, and reduced retinal ganglion cell dendritic motility with increased dendritic density (Xu et al., 2010). Ocular dominance plasticity during development was enhanced in mice lacking PirB, an innate immune receptor that binds MHC-I, or mice lacking H2-Kb and H2-Db, the two classical MHC-I αchains of C57BL/6 mice (Datwani et al., 2009). Mice that lacked H2-Db and H2-Kb also showed defects in synapse elimination and formation of eye-specific layers in visual processing areas of the brain, which was rescued by restoring H2-Db expression selectively in CNS neurons (Lee et al., 2014). Interestingly, in retina-thalamic co-cultures, a soluble form of MHC-I inhibited retinal outgrowth to thalami that expressed high neuronal MHC-I (Washburn et al., 2011).

There is also evidence that neuronal MHC-I acts to regulate synapses in the hippocampus. An initial report (Corriveau et al., 1998) showed that MHC-I expression was increased by seizure. Later reports showed a variety of effects on synaptic plasticity. In mice deficient for the TCR subunit CD3zeta, hippocampal long-term potentiation was enhanced and long-term depression was absent (Huh et al., 2000). Other reports indicate that neuronal MHC-I inhibits NMDAR function (Fourgeaud et al., 2010) and is critical for hippocampus-dependent memory (Nelson et al., 2013). These effects may in part be due to altered synaptic morphology, as hippocampal neurons with high levels of MHC-I maintain accelerated neurite outgrowth and polarization with more primary neurites (Bilousova et al., 2012). There thus appears to be a number of interacting pathways that can be influenced by MHC-I expression in hippocampal synapses.

Neuronal MHC-I expression has also been shown to regulate long-term depression and limit motor learning in cerebellum (McConnell et al., 2009) and the density and function of cortical synapses *in vitro* and *in vivo* (Glynn et al., 2011).

## Roles for MHC-I in axonal regeneration

Neuronal MHC-I expression is further implicated in models of axonal regeneration. Peripheral nerve transection in MHC-I knockout mice resulted in more extensive detachments from presynaptic terminals from perikarya and dendrites of axotomized neurons than wild-type animals (Oliveira et al., 2004). These results suggest that MHC-I molecules regulate the ability of neurons to regenerate axons.

A subsequent study found that in mice with strong axonal regrowth potential, axotomy produced a pronounced upregulation of MHC-I in spinal cord and a rapid loss of afferents, but that C57BL/6J mice, which exhibit poor axonal regenerative potential, displayed less MHC-I increase and a slower stripping of the synapses. These results suggest that neuronal expression of MHC-I during the first week after lesion enhances axonal regeneration (Sabha et al., 2008), and support the observation that elevated neuronal MHC-I expression promotes the recovery of locomotor abilities after spinal cord injury (Joseph et al., 2011). It has thus been suggested that MHC-I and MHC-I receptors may provide new targets to promote neurorepair following injury (Wu et al., 2011).

Previous data suggest that enhanced levels of neuronal MHC-I facilitate axonal regeneration, although it is still unclear how this molecule might help axons to recover after a lesion. Shatz (2009) have shown that PirB, an immune receptor that binds MHC-I, is highly expressed in neurons of particular brain regions, including the cerebral cortex, the olfactory bulb and the cerebellum. PirB is also located in growth cones and axons of cerebral cortical neurons *in vitro* (Syken et al., 2006). It has been proposed that MHC-I is located postsynaptically near glutamate receptors, whereas PirB is present presynaptically in axonal growth cones of cortical neurons *in vitro*. In that model, PirB would signal when bound to MHC-I located across the synapse. Since neural activity regulates MHC-I expression levels, PirB could also regulate downstream signaling cascades in an activity-dependent manner (Shatz, 2009). This sequence of events may explain why an increase of MHC-I/PirB molecules facilitates axonal regeneration.

An alternate explanation is based on a recent report on Cx3cr1, a chemokine receptor highly expressed in microglia (Wolf et al., 2013). Cx3cr1 deficiency causes a transient reduction of microglia during the early postnatal period and a consequent deficit in synaptic pruning, which is associated with weak synaptic transmission and decreased functional brain connectivity (Zhan et al., 2014). A lack of neuronal MHC-I could be related to a decrease of microglia, while increased neuronal MHC-I could promote a higher number of microglial cells that according to Zhang et al. (2014) could improve synaptic pruning and recovery of axons after a lesion.

## Is neuronal MHC-I immunologically functional?

During a period when neurons were generally regarded as MHC-I deficient, Medana et al. (2000) identified immunological functions of MHC-I in cultured neurons. They induced MHC-I and Fas receptor in murine hippocampal neurons with IFN-γ: the Fas receptor promotes apoptosis when it interacts with Fas ligand, a type-II transmembrane protein on T cells (Wajant, 2002; Figure 1). The MHC-I positive neurons were then challenged with the peptide GP33, an epitope of the lymphocytic choriomeningitis virus envelope glycoprotein, and with alloreactive CTLs to GP33. The MHC-I-expressing neurons pulsed with GP33, but not a control peptide, were killed by GP33-specific CTLs in a manner that did not require perforin, a pore-forming cytolytic protein released from CTLs granules (Tschopp et al., 1986), but did require Fas/FasL (Medana et al., 2000). A subsequent study reported that perforin, however, can play a role in CTL/neuron interactions by silencing neuronal activity prior to cell death (Meuth et al., 2009). Together, the Medana and Meuth studies introduced the hypothesis that MHC-I expressing neurons could be selectively targeted and destroyed by T cells.

## Roles for neuronal MHC-I in viral mediated neuroinflammation, brain disease and neurodegeneration

Pereira and Simmons (1999) showed that the H2 heavy chain and β2m components of the MHC-I molecule were both present on the surface of primary sensory neurons within 1–2 weeks after herpes simplex virus infection; some of these neurons were in close proximal association with T cells *in vivo*, suggesting a possible immunological interaction between neuronal MHC-I and T cells. Such an interaction was recently demonstrated as brain-isolated CTLs were found to destroy neurons infected with the neurotropic Borna disease virus in an antigen- and MHC-I dependent manner. Neuronal apoptosis were detected only hours after initial contact (Chevalier et al., 2011). Together, these studies indicate that the virus-specific CTLs can act as immune effectors in CNS viral infections.

The identification of neuronal MHC-I expression in human is relatively recent, starting with a report on a childhood viral infection, Rasmussen’s encephalitis (Bien et al., 2002). In human autopsy, immunolabel for the MHC-I component, β2m, was identified in cortical and hippocampal neurons, and CTLs were found in close apposition to these neurons. Analysis of the T cells demonstrated that there were clonal expansions of a subset of CD8^+^ T cells, but that the distribution CD4^+^ cells was normal. Granzyme B, a T cell cytotoxic molecule, was observed in CTLs in close appositions to neurons and astrocytes (Schwab et al., 2009). Together, these data strongly support antigen-driven MHC-I restricted, CTL-mediated attack against neurons and astrocytes in Rasmussen’s encephalitis.

Recently, a strong upregulation of neuronal MHC-I was reported in focal glioneuronal lesions associated with intractable epilepsy. This induction of MHC-I in neuronal cells may also be a feature of type II focal cortical dysplasia, tuberous sclerosis complex and ganglioglioma (Prabowo et al., 2013).

Another recent study suggests that human LilrB2, an immune cell receptor that binds MHC class I molecules and inhibits immune response (Barrow and Trowsdale, 2008), is a β-amyloid receptor, and that its murine homolog PirB regulates synaptic plasticity in an Alzheimer’s disease rodent model (Kim et al., 2013).

Multiple sclerosis (MS) has been linked to MHC-I and CTLs in the CNS (Friese and Fugger, 2005). Axonal injury and loss is a central determinant of irreversible neurological deficit and disease progression in patients with MS, and axon injury is most prominent within active, inflammatory demyelinated MS lesions enriched in CTLs (Bjartmar and Trapp, 2001). A recent study shows that axons are injured by antigen-specific CTLs through a MHC-I and granzyme B-dependent mechanism (Sauer et al., 2013), and suggests that CTLs may provide therapeutic targets in MS.

Our findings in human postmortem samples of adult control individuals and PD patients show that MHC-I is expressed by SN DA and LC NE neurons (Cebrián et al., 2014) which may have further implications for neurodegeneration. The proportion of catecholamine LC neurons that expressed MHC-I in humans was higher in controls than in PD subjects, which suggests that neurons with MHC-I expression may be more prone to cell death during the disease. The preference for MHC-I expression by catecholamine neurons was replicated in cultured SN DA murine neurons in which MHC-I was induced by IFN-γ and microglia activated by NM or α-syn, substances found extracellularly in postmortem PD brain, or by chronic exposure to the DA precursor, L-DOPA, which may be related to intracellular oxidative stress due to high cytosolic levels of its metabolite, DA. We found that cultured SN murine neurons can process and present foreign protein antigens by MHC-I, and that in the presence of the appropriate antigen and CTLs, the neurons are killed by CTLs (Cebrián et al., 2014). These findings suggest that neuronal MHC-I expression and antigen display by catecholamine neurons can be triggered by microglial activation or high cytosolic DA, features thought to be typical of PD, and that in the presence of the appropriate antigen and CTLs, MHC-I could play a role in neuronal death in diseases with robust CNS inflammation.

## Conclusions

Neuronal MHC-I expression plays multiple roles. First, it regulates synaptic plasticity during brain development. Second, it regulates axonal regeneration and the appropriate specification of synaptic inputs following injury. Third, in neuronal diseases including neurotropic viral infections, neuronal MHC-I expression is upregulated and may initiate T cell mediated responses. While current research in each of these areas is ongoing, the suggestion of a role in neurodegenerative disease is the most recent and the least understood: while there is a consensus that many neurodegenerative diseases feature a robust inflammatory response, it remains unclear how this is related to chronic disease processes.

Our recent study demonstrates neuronal MHC-I expression in both normal and PD adult brain; such expression to date appears to be particular for catecholaminergic/monoaminergic neurons. *In vitro* experiments indicate that DA primary human neurons derived from human embryonic stem cells and primary catecholamine murine neurons are more susceptible to MHC-I induction by IFN-γ than other neuronal populations (Cebrián et al., 2014), which may be related to unusually high oxidative stress in these neurons. The findings suggest that an immunologically-based mechanism may link activated microglia, increased cytosolic oxidative stress and neuronal death of catecholamine neurons in PD and other diseases of this system. For PD, microglia activated by NM, native α-syn, modified α-syn, or mutant α-syn release IFN-γ that in turn can induce MHC-I expression in these neurons. The capacity of catecholamine neurons to process and display antigens may thus render them selective targets for T cell mediated cell death.

These possibilities are consistent with recent demonstrations that microglia can be activated by substances released from degenerating neurons in PD, such as α-syn (Zhang et al., 2007; Béraud et al., 2013) or NM (Zhang et al., 2011, 2013c), and that activated microglia can elicit neurotoxicity (Block et al., 2007; Lull and Block, 2010; Zhao et al., 2013). Both NM and α-syn are found extracellularly in the postmortem brain of PD patients (Double, 2012), a disorder that features high levels of activated microglia in the SN (Foix and Nicolesco, 1925) and high levels of intracellular oxidative stress (Fahn and Sulzer, 2004). Parkinson’s disease patient brain features increased IFN-γ (Mogi et al., 2007) and chemokines (Harris et al., 2012), as well as a compromised blood brain barrier (Farkas et al., 2000; German et al., 2012) that may explain why CTLs are substantially higher in PD patients than age-matched controls (Hisanaga et al., 2001; Brochard et al., 2009). CD4+ T helper cells have also been shown to infiltrate the brain in human PD postmortem samples and exert a cytotoxic effect in mouse brain following nigrostriatal injury (Brochard et al., 2009); thus, T cells and antigen presentation, as well as activated microglia could play a role in PD pathogenesis.

Neuronal display of antigenic MHC-I could participate in a range of additional neurological disorders. For example, Japanese encephalitis virus can induce MHC-I expression in non-neuronal cells by interferon type 1 (Abraham et al., 2010), while in mice, IFN-γ plays a role in paraquat-induced neurodegeneration (Mangano et al., 2012). Central nervous system-directed expression of IFN-γ produces basal ganglia calcification and nigrostriatal degeneration (Chakrabarty et al., 2011). In human case studies, a link is described between chronic hepatitis C patients who were treated with type 1 interferon and developed PD-like symptoms that reversed when the treatment was halted (Almeida et al., 2009).

**Figure 2:**
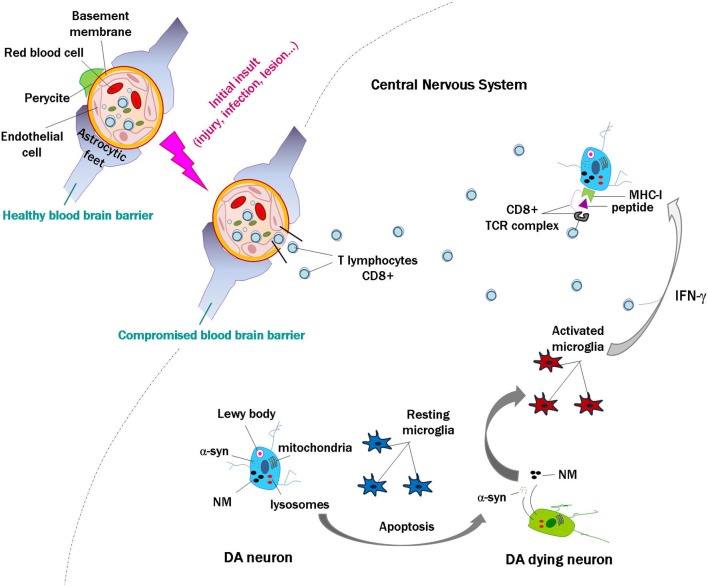
**Scheme modeling an initial insult and disruption of the blood brain barrier, with penetration of lymphocytes into the brain**. In the event of death of a few catecholamine neurons, alpha-synuclein (α-syn) and neuromelanin (NM) are released to the extracellular space, activating microglial cells that in turn release proinflammatory substances such as interferon gamma (IFN-γ). This would induce major histocompatibility class I expression (MHC-I) in the membrane of catecholamine neurons, and if they contain proteins that are misfolded or cannot be normally degraded, these might be presented as antigens by MHC-I. Cytotoxic lymphocytes that enter the brain may recognize these presented antigens and kill the neurons, which would again release α-syn and NM. This could continue a vicious cycle that may contribute to the death of catecholamine neurons in Parkinson’s disease.

Together, these data indicate that in human brain, neuronal MHC-I expression, antigen presentation and the presence of T cells could occur simultaneously under certain circumstances, leading to the death of targeted neurons. In some neurodegenerative diseases, and in particular for PD, we propose that activation of lymphocytes in the periphery may occur in response to self or non-self proteins, or as an initial insult and disruption of the blood brain barrier, with a subsequent penetration of lymphocytes in the brain. When catecholamine neurons die with subsequent release of α-syn and NM to the extracellular space, the activation of microglial cells will release proinflammatory substances such as IFN-γ, leading to an upregulation of MHC-I in the membrane of catecholamine neurons that could present neuronally derived antigens. If lymphocytes are close, they could recognize these antigens and target and kill the cell, which would again release α-syn and NM (Figure 2), leading to a vicious cycle that would enhance with time the neuronal death and pathogenesis of PD.

Future studies are necessary to identify which antigens are presented by MHC-I expressing catecholamine neurons and how T cells might interact with them. If these interactions occur, immune therapies used in other diseases including classical autoimmune disorders such as Type 1 diabetes or MS may be adapted to provide future treatments for PD.

## Conflict of interest statement

The authors declare that the research was conducted in the absence of any commercial or financial relationships that could be construed as a potential conflict of interest.
